# Job strain and ischemic heart disease: the balance of methodological bias and implications for prevention. Response to: Bonde JP et al. The demands–control–support work stress model and risk of ischemic heart disease: causal inference based on observational epidemiology.

**DOI:** 10.5271/sjweh.4315

**Published:** 2026-09-01

**Authors:** Mathilde Lavigne-Robichaud, Paul Landsbergis, Chantal Brisson, Grace Sembajwe, Mahée Gilbert-Ouimet, Jian Li, Alain Milot, Xavier Trudel

**Affiliations:** 1 Centre de recherche du CHU de Québec–Université Laval (CRCHUQ–ULaval), Québec, Canada.; 2 SUNY Downstate School of Public Health, Brooklyn, NY, USA.; 3 Indiana University School of Public Health - Bloomington, Indianapolis, IN, USA.; 4 Inserm, Institut de recherche en santé, environnement et travail (Irset) - UMR_S 1085, Univ Angers, CHU Angers, Univ Rennes, EHESP, SFR ICAT, Angers, France.; 5 Université du Québec à Rimouski (UQAR), Rimouski, Canada.; 6 Departments of Environmental Health Sciences and Epidemiology, Fielding School of Public Health, Joe C. Wen School of Nursing, Univeristy of California Los Angelse, Los Angeles, CA, USA.; 7 Department of Medicine, Université Laval, Québec, Canada.; 8 Department of Social and Preventive Medicine, Université Laval, Québec, Canada.

We read with interest Bonde et al’s (1) recent review. We agree with their premise: strengthening causal inference is an important objective for occupational epidemiology. However, we believe the conclusion that “at most, any true effect [of job strain on ischemic heart disease (IHD)] appears to be small” is not supported by a valid appraisal of the available evidence. The pooled relative risk estimate (RRE) of 1.14 is most likely underestimated, as common limitations in the available literature tend to bias results toward the null.

The authors acknowledge underestimation sources: nondifferential exposure misclassification, overadjustment for cardiometabolic risk factors, and healthy-worker survivor selection (1). Additional sources of underestimation are not discussed. Dichotomizing exposure by combining active and passive exposures into a single “non–high-strain” category may attenuate risk estimates by increasing heterogeneity in the reference group. Indeed, workers with passive exposure may also be at increased IHD risk (2). The pooled RRE may further be attenuated by sex: women develop IHD at older ages partly due to pre-menopausal estrogen cardioprotection, thus working-age follow-up captures fewer events, reducing pooled estimates and precision (3).

Sources of overestimation raised also warrant closer scrutiny. For instance, lower estimates in job-exposure matrix (JEM) studies are interpreted as evidence of upward bias in self-reported studies. However, even when exposure values are imputed within subgroups defined by sex and age, JEM do not fully capture individual-level variability in exposure within occupational categories (4). The resulting non-differential misclassification likely attenuates estimates, a limitation the authors acknowledge but do not take into account in their conclusion. The supporting reference for overestimation relies on a 4-item measure of perceived stress (5), limiting its relevance to job strain. An additional source of overestimation is negative affectivity, in which adverse health perceptions inflate individual-level exposure reports. However, this mechanism is not supported in prospective studies with control for anger, hostility, and cynicism (6, 7). Finally, the authors raise concerns about a health-reporting bias: workers with prodromal IHD symptoms may over-report perceived job strain, inflating observed associations. In prospective studies excluding early incident IHD events, associations were not attenuated and, if anything, marginally strengthened (6, 8), providing no support for reverse causation as a source of overestimation. More broadly, methodological characteristics are presented as isolated binary indicators rather than as interdependent dimensions. This hinders the overall appraisal of study quality.

These methodological considerations have implications for burden estimation. There has been considerable debate about whether the population attributable fraction (PAF) of 3.4% that the IPD-Work Consortium reported for job strain and IHD (8) was an underestimate when accounting for exposure misclassification, alternative referent group definitions, and other sources of attenuation identified in the literature (9, 10). A subsequent prospective cohort study designed to address several of the sources of underestimation discussed here estimated that 18.2% of incident IHD were attributable to job strain exposure (11).

In sum, while we share Bonde et al’s emphasis on causal inference, the balance of methodological bias in this literature is more plausibly downward than unpredictable. Given the substantial burden of IHD, debates about the precise magnitude should not delay the development and evaluation of workplace interventions to reduce job strain and improve cardiovascular health.
